# MicroRNA-Mediated Regulation of *ITGB3* and *CHL1* Is Implicated in SSRI Action

**DOI:** 10.3389/fnmol.2017.00355

**Published:** 2017-11-02

**Authors:** Keren Oved, Luba Farberov, Avial Gilam, Ifat Israel, Danielle Haguel, David Gurwitz, Noam Shomron

**Affiliations:** Faculty of Medicine, Sagol School of Neuroscience, Tel Aviv University, Tel Aviv, Israel

**Keywords:** *ITGB3*, *CHL1*, miR-221, miR-222, miR-151a-3p, selective serotonin reuptake inhibitors

## Abstract

**Background:** Selective serotonin reuptake inhibitor (SSRI) antidepressant drugs are the first-line of treatment for major depressive disorder (MDD) but are effective in <70% of patients. Our earlier genome-wide studies indicated that two genes encoding for cell adhesion proteins, close homolog of L1 (*CHL1*) and integrin beta-3 (*ITGB3*), and microRNAs, miR-151a-3p and miR-221/222, are implicated in the variable sensitivity and response of human lymphoblastoid cell lines (LCL) from unrelated individuals to SSRI drugs.

**Methods:** The microRNAs miR-221, miR-222, and miR-151-a-3p, along with their target gene binding sites, were explored *in silico* using miRBase, TargetScan, microRNAviewer, and the UCSC Genome Browser. Luciferase reporter assays were conducted for demonstrating the direct functional regulation of *ITGB3* and *CHL1* expression by miR-221/222 and miR-151a-3p, respectively. A human LCL exhibiting low sensitivity to paroxetine was utilized for studying the phenotypic effect of *CHL1* regulation by miR-151a-3p on SSRI response.

**Results:** By showing direct regulation of *CHL1* and *ITGB3* by miR-151a-3p and miR-221/222, respectively, we link these microRNAs and genes with cellular SSRI sensitivity phenotypes. We report that miR**-**151a-3p increases cell sensitivity to paroxetine via down-regulating *CHL1* expression.

**Conclusions:** miR-151a-3p, miR-221/222 and their (here confirmed) respective target-genes, *CHL1* and *ITGB3*, are implicated in SSRI responsiveness, and possibly in the clinical response to antidepressant drugs.

## Introduction

Major depressive disorder (MDD) is a complex, relatively common, costly, and recurrent mental disorder that, according to the World Health Organization, is the leading cause of disability worldwide and is among the leading causes of disease burden globally (Centers for Disease Control Prevention (CDC), 2010; Greenberg et al., 2015)^1^. One of the major risks of MDD is suicide, which has been reportedly linked to psychiatric disorders in general and to MDD specifically (Zweig and Hinrichsen, 1993; Lesage et al., 1994). Improving the treatment for MDD would have huge financial consequences and would greatly improve the quality of life for millions of patients globally. Selective serotonin reuptake inhibitor (SSRI) antidepressant drugs, which block serotonin uptake via binding directly to the serotonin transporter (SERT) (Sangkuhl et al., 2009), have remained for the past three decades as the first-line treatment for MDD (Thaler et al., 2012). However, about 30–40% of MDD patients fail to reach sufficient remission with SSRI treatment (Souery et al., 2007). In such cases, clinicians often increase the dosage of the same SSRI drug, switch to another antidepressant of the same or a different class [such as serotonin-norepinephrine reuptake inhibitors (Girardi et al., 2009), tricyclic antidepressants, or serotonin ligands (Ruhé et al., 2006)], or augment the antidepressant by adding the mood-stabilizing drug lithium (Price et al., 1990; Bauer et al., 2003).

Clinical guidelines recommend waiting at least 4–6 weeks before switching to an alternative drug (Kato and Serretti, 2010). Meanwhile, patients may experience long periods of depressive symptoms and an increased risk of suicide with no benefit from their first-line SSRI treatment. However, to date no diagnostic tools for predicting patient response to specific antidepressants are available (Dale et al., 2015).

Biomarkers for predicting antidepressant drug response, in particular for SSRIs as the first-line antidepressants, are therefore needed for aiding clinicians in drug and dosage choice in order to decrease the time from diagnosis to remission for the SSRI non-responder patient population. Additionally, reliable diagnostics are needed for early identification of treatment-resistant depression (TRD, patients who do not respond to any of the approved antidepressants). Several genome-wide association studies (GWAS) have searched for common single nucleotide polymorphisms (SNPs) associated with SSRI drug response (Ising et al., 2009; Garriock et al., 2010; Uher et al., 2010; Ji et al., 2013; Biernacka et al., 2015). However, none of these GWAS findings could be replicated, and several meta-analysis studies have been published (Tansey et al., 2012; GENDEP Investigators, MARS Investigators, STAR^*^D Investigators, 2013; Biernacka et al., 2015), which concluded that none of the identified SNPs had genome-wide significance.

A major landmark in mRNA regulation and protein expression levels came about with the discovery of microRNAs (miRNAs). miRNAs are short (22 nucleotides on average) endogenous non-coding RNAs that down-regulate gene expression at the post-transcriptional level (Bartel, 2009; Friedman et al., 2009; Shomron, 2009; Rukov and Shomron, 2011). Thousands of miRNAs are encoded within the human genome; they are prevalent in all cells, tissues (Liang et al., 2007) and body fluids (Weber et al., 2010; Gurwitz, 2015). At least half of all human gene transcripts are estimated to be targets of evolutionarily conserved miRNA regulation (Lewis et al., 2005; Friedman et al., 2009). Many miRNAs were implicated in various diseases including those involved in brain disorders (Mor et al., 2013; Serafini et al., 2014; Modai and Shomron, 2016).

Given that miRNA can potentially target dozens of genes, they have been recognized as master regulators of gene expression in multicellular organisms. Thus, changes in miRNA levels can affect and might even predict changes in global gene expression (Lim et al., 2005). Consequently, miRNAs are being studied as diagnostics, prognostics, therapeutics, or as pharmacogenomic biomarkers. Indeed, in recognition of their important role in health, disease, and drug response, a new trend in molecular medicine, termed “miRNA pharmacogenomics,” has emerged (Shomron, 2010; Rukov and Shomron, 2011; Rukov et al., 2011, 2014).

### Genome-wide expression profiling for identifying SSRI response biomarkers

We recently utilized human lymphoblastoid cell lines (LCLs) from unrelated healthy individuals for conducting a genome-wide transcriptomic microarray-based search for SSRI sensitivity and response to chronic treatment biomarkers (Morag et al., 2011; Oved et al., 2012, 2013). After applying this genome-wide, hypothesis-free approach, we reported several genes and miRNAs as tentative SSRI response biomarkers potentially implicated in the mode of action of SSRI antidepressants (Morag et al., 2011; Oved et al., 2012, 2013). Among these, the expression levels of *CHL1* (close homolog of L1) and miR-151a-3p, predicted by bioinformatics tools to target *CHL1*, were found to be associated with SSRI sensitivity (Morag et al., 2011; Oved et al., 2012). In a separate study, *ITGB3* (coding for integrin beta-3, also known as platelet glycoprotein IIIa and CD61) as well as miR-221 and the closely related miR-222, both predicted by bioinformatics tools to target *ITGB3*, exhibited the most consistent expression level changes following chronic (21 days) paroxetine exposure of human LCLs (Oved et al., 2013). Additionally, *ITGB3* and miR-221/miR-222 exhibited opposite expression level changes (Oved et al., 2013). Both *CHL1* and *ITGB3* code for cell adhesion proteins implicated in neurogenesis and synaptogenesis, and therefore seem to be promising SSRI response biomarkers. Notably, *CHL1* knockout mice exhibit mood-related neurological deficits as well as a defective organization of the limbic serotonergic neurons, projecting from thalamic nuclei to the visual rather than prefrontal cortex (Buhusi et al., 2003; Montag-Sallaz et al., 2003; Demyanenko et al., 2004, 2010, 2011; Carneiro et al., 2008; Cingolani and Goda, 2008; Cingolani et al., 2008; Schlatter et al., 2008; Carter et al., 2011; Huang et al., 2011; Katic et al., 2014; Kleene et al., 2015; Mazalouskas et al., 2015). Indeed, cell adhesion proteins, including those coded by *CHL1* and *ITGB3*, were shown to play key roles in neurogenesis and synaptogenesis, which in turn, are crucial for remission from depression (Thomas and Peterson, 2008; Hanson et al., 2011; Danzer, 2012; Eisch and Petrik, 2012; Eyre and Baune, 2012; Bambico and Belzung, 2013; Mateus-Pinheiro et al., 2013; Duman, 2014; Duman and Duman, 2015).

In order to link the genes and miRNAs (Morag et al., 2011; Oved et al., 2012, 2013) to a single set cohort of potential SSRI response biomarkers, we integrated our findings and identified five candidate miRNA-target gene pairs (see Methods). miR-221/222, miR-151a-3p, and their predicted target genes, *ITGB3* and *CHL1*, respectively, were chosen for further studies, since they were implicated in our proposed model regarding the mode of action of SSRI drugs (Oved et al., 2013). This model depicts the cell membrane proteins encoded by *CHL1* and *SLC6A4* (coding for the serotonin transporter), competing on a limited cell membrane protein pool of integrin beta-3 (encoded by *ITGB3*) (Oved et al., 2013).

The aim of the current study was to show the direct regulation of *CHL1* and *ITGB3* by their proposed regulators, miR-151a-3p and miR-221/222, respectively. In addition, we examined the phenotypic effects of altering the expression of these genes on the *in vitro* SSRI sensitivity of cultured human cells. Furthermore, this study also explored the expression levels of candidate genes and miRNAs that we previously reported as associated with SSRI response and that are known to be implicated in cell adhesion, in LCLs cultured in serum-free (SF) compared with serum-supplemented media. The adhesion of cultured cells to the matrix is known to be enhanced in the absence of serum (Thirumala et al., 2007; Audiffred et al., 2010; Nakayama et al., 2014).

Our new findings link miR-151a-3p and miR-221/222 with SSRI sensitivity phenotypes in human cells *via* direct regulation of *CHL1* and *ITGB3*. Importantly, we show that human miR-151a-3p and miR-221/222 and their respective target genes, *CHL1* and *ITGB3*, may be implicated in the response of human LCLs to SSRI antidepressant drugs and may tentatively serve as novel MDD drug targets, following validation by additional studies using clinical blood samples and/or studies with brain tissues or blood samples from animal MDD models.

## Methods

### Data integration analysis for miRNAs and their target sites

**Figure 1:**
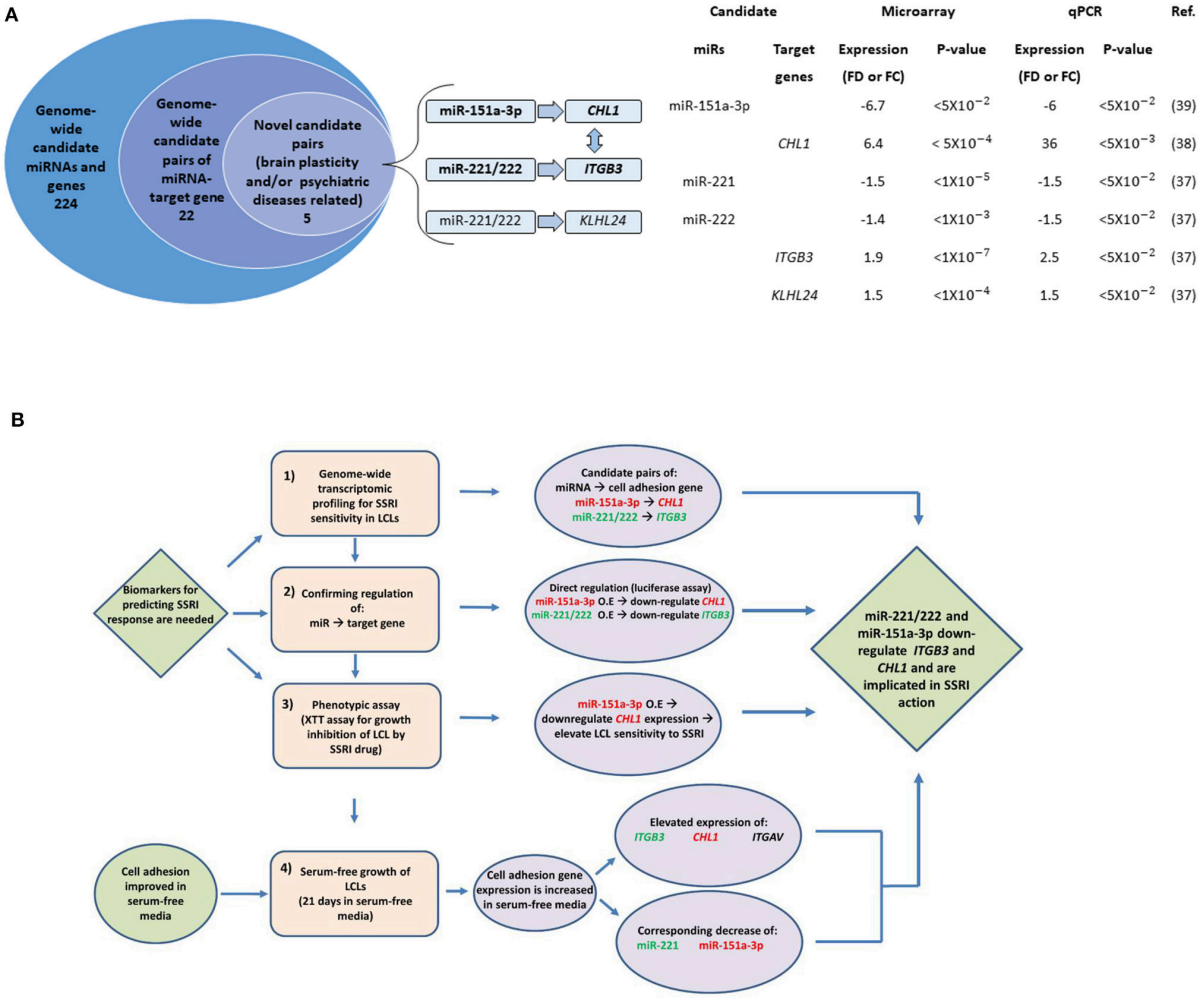
The study design. **(A)** Data integration findings from our previously published genome-wide transcriptomic studies (Morag et al., 2011; Oved et al., 2012, 2013). On the left: A stacked Venn diagram. The outer circle represents the number of candidate miRNAs and genes showing a fold-difference>1.4 and a statistical significance of *p* < 0.05. The second inner circle represents the number of predicted miRNA-target gene pairs using TargetScan and additional software tools (see Oved et al., 2012, 2013). The third inner circle represents the number of miRNA-target gene pairs for which both miRNA and the target gene were reportedly expressed in neuronal cells and were associated with brain plasticity (synaptogenesis/neurogenesis) or with psychiatric diseases (note that the five pairs include both miR-221 and miR-222). The horizontal arrows denote the relationship between miRs and target genes. The vertical arrow denotes a mutual physical interaction between ITGB3 and CHL1 taking place in the cell membrane (Katic et al., 2014). On the right: Microarray and qPCR data for candidate miRNA-target gene pairs. Fold-difference represents basal expression levels in LCLs with low vs. high paroxetine sensitivity. Fold-change represents expression levels in LCLs following paroxetine exposure compared with controls grown and studied in parallel, as measured by microarray and qPCR experiments. **(B)** A flow chart presenting the study design. Phase 1 represents the results generated from previous studies described in Figure 1A. Abbreviation: FD, Fold difference. FC, Fold change. O.E., Over-Expression.

The microRNAs miR-221, miR-222, and miR-151-a-3p, along with their binding sites, were explored *in silico* using miRBase (Griffiths-Jones et al., 2008)^3^, TargetScan^4^, microRNAviewer (Kiezun et al., 2012)^5^, and the UCSC Genome Browser (Multiz Alignment of 100 Vertebrates)^6^ In compiling the data in Figure 1, we first compiled a list of 224 candidate biomarker SSRI response genes and miRNAs identified in our earlier studies using genome-wide searches (Morag et al., 2011; Oved et al., 2012, 2013). We then created a list of the top 22 predicted miRNA-target gene pairs using TargetScan and additional software tools, based on miRNA-binding site conservation and a high level of agreement between different software (Oved et al., 2012, 2013). Next, we screened for novel miRNA-target gene pairs for which, both miRNA and the target gene, were reportedly expressed in neuronal cells and were associated with brain plasticity (synaptogenesis/neurogenesis) or with psychiatric diseases; we identified five such pairs.

### Cell culture

Human LCLs were obtained from the National Laboratory for the Genetics of Israeli Populations (NLGIP) at Tel-Aviv University as described in Morag et al. (2010, 2011); Oved et al. (2012, 2013)^7^. The cell lines were immortalized from the peripheral blood lymphocytes of consenting healthy adult donors. Cells were maintained in RPMI medium supplemented with 10% FBS and antibiotics (100 U ml−1 penicillin; 100 μg ml−1 streptomycin) and kept at a temperature of 37°C, with 6% CO2 and 100% humidity. The human cell lines MCF-7 (adenocarcinoma breast cell line), HEK-293T (transformed human embryonic kidney cells), and SH-SY5Y neuroblastoma cells were maintained in DMEM medium supplemented with 10% FBS and antibiotics (100 U ml−1 penicillin; 100 μg ml−1 streptomycin) under similar conditions.

### Serum-free growth of LCLs

LCLs previously maintained in 10% FBS-containing medium were washed in PBS and then resuspended in serum-free RPMI medium containing 4% BIOGRO-2 (Biological Industries, Israel) commercial serum supplement. This BIOGRO-2 concentration was previously reported to be optimal for long-term serum-free growth of LCLs (Milanesi et al., 2015). Control cultures were grown in parallel with 10% FBS.

### miRNA constructs

The miRNA expression vectors, miRVec-221 and miRVec-222, which contain the genomic regions of human pre-miR-221 and pre-miR-222, respectively, were provided by Prof. Reuven Agami (Voorhoeve et al., 2006). The genomic region of the human pre-miR-151a was cloned into the BamHI–EcoRI restriction sites of the miRNA expression vector miRVec. miRVec-151a-3p was prepared with the genomic loci ~70 bp upstream and downstream of the pre-miR by PCR-amplification from human genomic DNA (gDNA). BamHI–EcoRI restriction sites were added (indicated by uppercase letters) to the primers:

miRVec-151a-3p forward: gcGGATCCgctaaactaaccctcctgtcagccc
miRVec-151a-3p reverse: gccttGAATTCagtgcctgggtgactcttcctg

### Dual luciferase reporter assays

Fragments of ~500 bp from *CHL1* and *ITGB3* 3′UTR, spanning the miRNA-binding sites, were cloned into the XhoI–NotI restriction site downstream of the Renilla luciferase reporter of the psiCHECK-2 plasmid (Promega, USA) that contains a Firefly luciferase reporter (used as a control) under a different promoter. For this purpose, the 3′ UTR fragments were PCR amplified using Phusion High-Fidelity DNA Polymerase (Finnzymes) from gDNA of LCL, and XhoI–NotI restriction sites were added. The miRNA binding sites were mutated using the QuikChange Lightning Site-Directed Mutagenesis Kit (Agilent, USA). For luciferase assays, HEK-293T, MCF-7, and SH-SY5Y cells were transfected using Lipofectamine 2000 transfection reagent. Next, the cells were transfected with 5 ng of psiCHECK-2 plasmid containing the desired 3′ UTR, with or without site-directed mutations, and 485 ng miRVec containing the desired pre-miRNA or an empty vector. At 24 and 48 h after transfection, firefly and Renilla luciferase activities were measured using the Dual Luciferase reporter assay system kit (Promega, USA) and the LUMIstar Omega Luminometer (BMG LabTech, Germany), according to the manufacturer's recommendations. Renilla luciferase results were normalized to the values of the firefly luciferase. Results represent 3–4 biological replicates. Transfection efficiencies were measured by Green Fluorescent Protein (GFP) fluorescence measurements in all cells, indicating a reproducible transfection efficiency of at least 20%.

### miRNA transfections

For miRNA transfection experiments, LCL code #5000 was seeded in 12-well plates at a concentration of 9.6^*^10^5^ cells/well and transfected with 2 μg of miRVec-151a-3p or an empty vector (for 24 h), or in 24-well plates at a concentration of 4.8^*^10^5^ cells/well and transfected with 1 μg of the indicated vectors (for 6 and 12 h). Transfections were performed in triplicate using Lipofectamine 2000 transfection reagent (Invitrogen, USA) according to the manufacturer's instructions. Following 6, 12, or 24 h transfection, RNA was extracted. Results of miR-151a-3p over-expression and *CHL1* expression down-regulation represent three technical replicates. Transfection efficiencies were measured by GFP fluorescence in all cells, indicating a transfection efficiency of at least 20%.

### RNA extraction

Total RNA purification was achieved using phenol-chloroform extraction: cells were centrifuged and then lysed using TRIzol Reagent (Thermo Fisher Scientific, USA), followed by RNA separation using chloroform and isopropanol precipitation. The final RNA concentration and purity were measured using a NanoDrop ND-1000 spectrophotometer (NanoDrop Technologies, Thermo Fisher Scientific, USA).

### Cell proliferation assays

LCL code #5000 was seeded in 96-well plates at a concentration of 2^*^10^5^ cells/well. Following 12 h transfection with 200 ng of miRvec-151a-3p or empty vector, growth inhibition of LCL was examined by exposure to 10 μM paroxetine for 24 h. Following 24 h, XTT cell proliferation assays (Biological Industries, Israel) were carried out as previously described (Morag et al., 2010, 2011). Results represent 4 biological replicates.

### Real-time RT-PCR

Reverse transcription reactions for mRNA and for specific mature miRNAs were performed using the High-Capacity cDNA Reverse-Transcription Kit with random primers or TaqMan miRNA assays, respectively, according to the manufacturer's recommendations (Life Technologies, USA). The expression of single miRNA or mRNA was tested similarly using TaqMan Universal PCR Master Mix (Life Technologies, USA) or Solaris qPCR Gene Expression Master Mix (Thermo Scientific, USA), respectively as described (Oved et al., 2012, 2013), and the specific Solaris quantitative PCR gene-expression assay (Thermo Scientific, MA, USA) or the ABI TaqMan Assay probe (ABI, USA). PCR amplification and analysis were performed using the Step-One Detection System (ABI, USA). Comparative critical threshold (Ct) values, obtained by real-time PCR analysis, were used for relative quantification of gene or miRNA expression and determination of the fold-change of expression. Fold changes were obtained by using the formula: 2^−ΔΔCt^ (Schmittgen and Livak, 2008). Individual forward and reverse primer sequences are detailed next:

**Table:** 

**Gene**	**Forward primer**	**Reverse primer**
GUSB (control)	CTGCTGGCTACTACTTGAAGATG	GAGTTGCTCACAAAGGTCAC
ITGB3	ACCAGTAACCTGCGGATTG	CAGGTGGTCTTCATATCATAGC
CHL1	GCACAGCCAGCAATTTCTTG	TCTTTGTCCAGCGAGGA
ITGAV	ABI assay ID - Hs00233808	
SLC6A4	ABI assay ID - Hs00984349	
**miRNA**	**Mature miRNA sequence**	**ABI assay ID**
U6 snRNA (control)	GTGCTCGCTTCGGCAG CACATATACTAAAATTG GAACGATACAGAGAAG ATTAGCATGGCCCCTG CGCAAGGATGACACGC AAATTCGTGAAGCGTT CCATATTTT	1973
miR-221 (miR-221-3p)	AGCUACAUUGUCUGCUGGGUUUC	524
miR-222 (miR-222-3p)	AGCUACAUCUGGCUACUGGGU	2276
miR-151a-3p (miR-151-3p)	CUAGACUGAAGCUCCUUGAGG	2254

## Results

### Regulation of *ITGB3* and *CHL1* transcription by miRNAs

**Figure 2:**
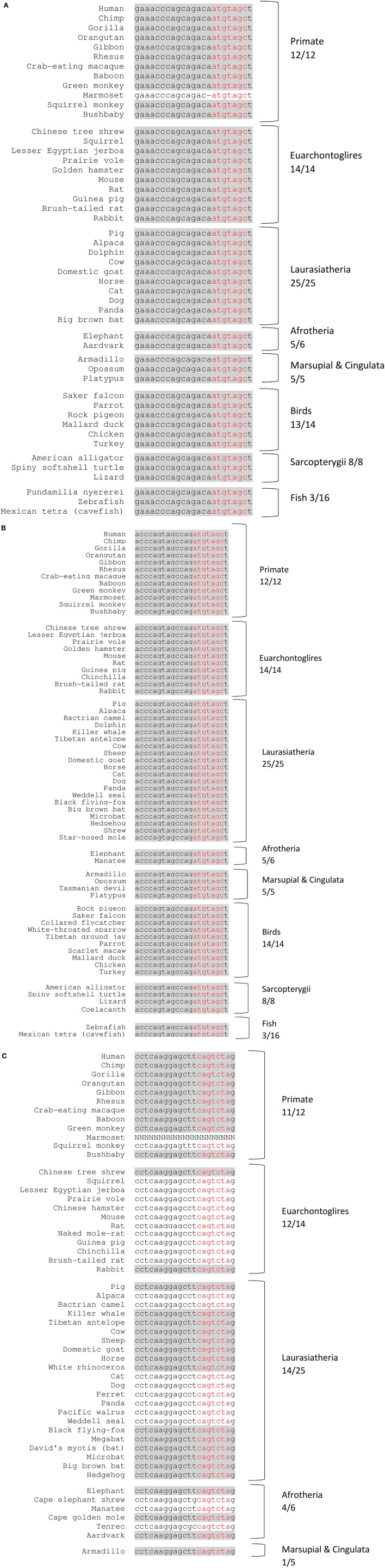
Conserved miRNAs across representative vertebrate species. Shading represents conserved fully mature miRNA. Red nucleotides represent the conserved seed region. x/y represents the number of species out of the total subset with a conserved seed region. **(A)** miR-221 (chr. X position 45746180–45746202 +strand. **(B)** miR-222 (chr. X position 45747036–45747056 +strand). **(C)** miR-151a-3p (chr. 8 position 140732587–140732607 +strand).

MicroRNAs, miR-221 and miR-222, which are clustered genes located in an intergenic region on the X chromosome in humans and have the same seed sequence, were found to be broadly conserved among vertebrates (89/100 with the conserved seed region), and highly conserved among primates (12/12 with the conserved mature miRNA region) (Figures 2A,B, and Supplementary Figures S1A,B). miR-151a-3p, which is located within intron-22 of the host gene PTK2 (also known as FAK) on chromosome 8 in humans, was found to be conserved only among mammals (52/62 with the conserved seed region), and especially among primates (11/12 with the conserved seed region) (Figure 2C and Supplementary Figure S1C).

**Figure 3:**
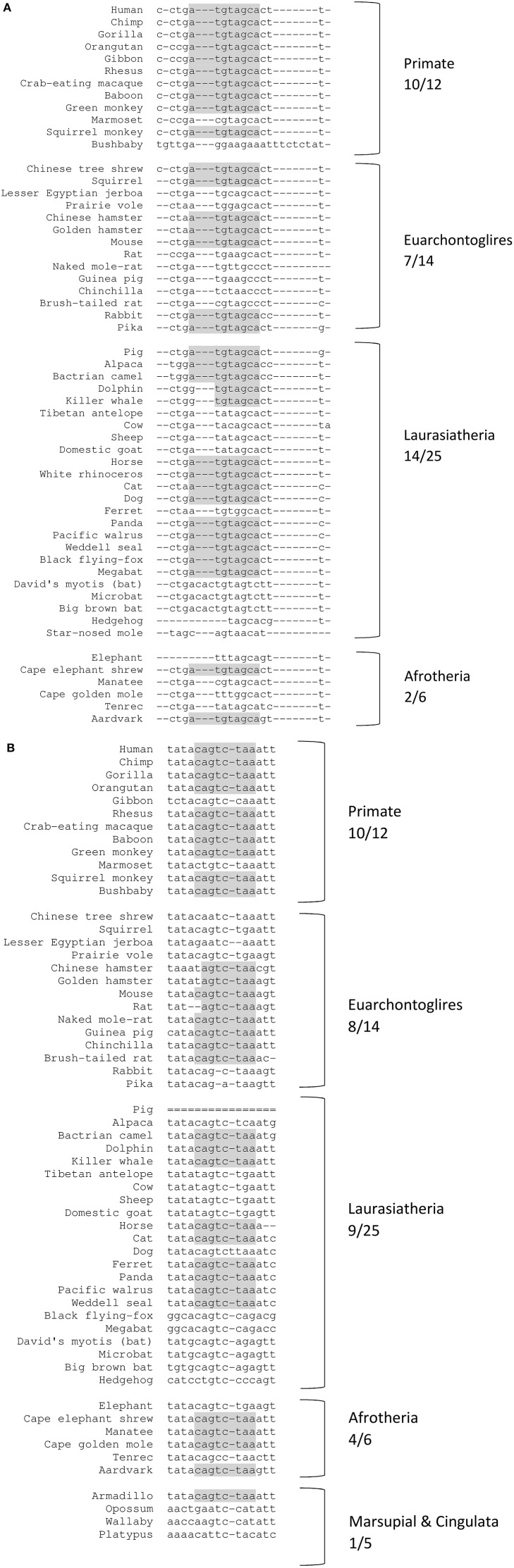
Conserved miRNA binding sites across representative mammal species. Shading represents conserved nucleotides – note: for the primate species, the binding sites are of the 8-mer type (the seed region + position 8), and are followed by an adenosine residue. x/y represents the number of species out of the total subset with the conserved miR binding site (seed region). **(A)** miR-221/222 target site at *ITGB3* 3′UTR (chr. 17 position 45389536-45389550). **(B)** miR-151a-3p target site at *CHL1* 3′UTR (chr. 3 position 447894-447908).

**Figure 4:**
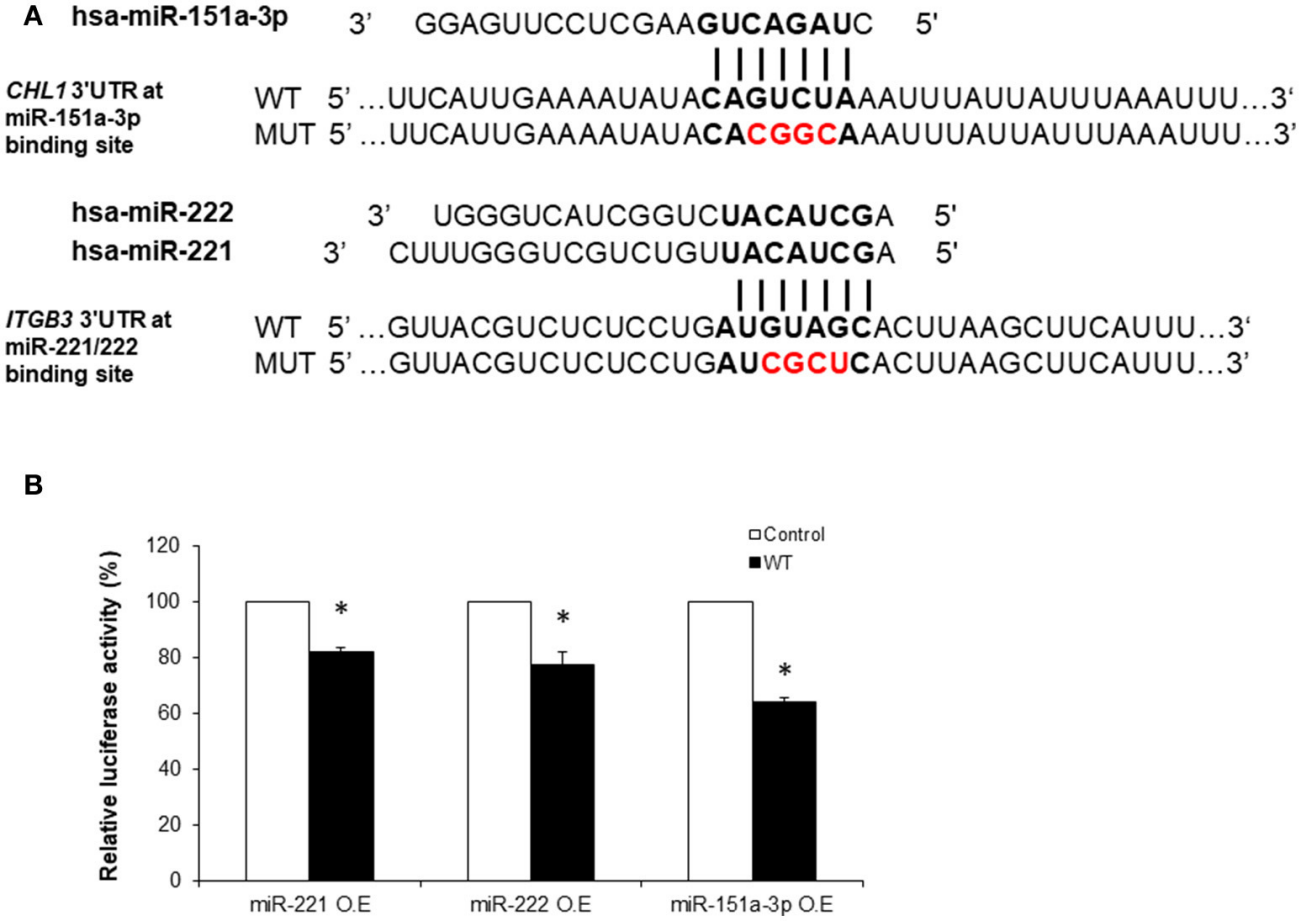
Luciferase reporter assays demonstrating the *in vitro* regulation of *ITGB3* by miR-221/222 and of *CHL1* by miR-151a-3p. **(A)** Sequences of Renilla/Firefly Luciferase psiCHECK2 constructs under the regulation of *ITGB3* and *CHL1* 3′UTRs that were used for transient reporter assay experiments. WT and Mutant alleles for miR-221/222 and miR-151a-3p binding sites are presented. **(B)** Luciferase activity 24 h following co-transfection with miR-221, miR-222 or miR-151a-3p combined with either of the *ITGB3* or *CHL1* 3′UTR constructs (WT vs. mutant) using the HEK-293T cell line. Values are presented as the % mean ± SEM (*n* = 3–4; ^*^p < 0.05). Bold nucleotides at the *ITGB3* and *CHL1* 3′UTR constructs represent the miRNA binding sites. Red nucleotides represent the 4 mutated nucleotides at the seed miR-221/222 or miR-151a-3p binding sites. O.E., Over-Expression.

For both human miR-221/222 and miR-151a-3p, the binding sites are of the 8-mer type, defined as an exact match to positions 2–8 of the mature miRNA, the seed region + position 8 and are followed by an adenosine residue (Figures 3, 4A). Having a canonical 8-mer site was shown to be important for recognition and increased down-regulation of the targeted gene (Brennecke et al., 2005; Grimson et al., 2007). Furthermore, these miRNA binding sites are well conserved across mammals (33/62 mammals for the miR-221/222-*ITGB3* binding site; 32/62 mammals for the miR-151a-3p-*CHL1* binding site), particularly primates (10/12 primates for the miR-221/222-*ITGB3* binding site and for the miR-151a-3p-*CHL1* binding site) (Figure 3 and Supplementary Figure S2).

In order to demonstrate the direct functional regulation of *ITGB3* and *CHL1* expression by miR-221/222 and miR-151a-3p, respectively, luciferase reporter assays were conducted as follows: a region of ~500 bp from the 3′UTRs of human *ITGB3* and *CHL1* genes, containing a tentative miRNA binding site, was cloned into the Renilla/Firefly Luciferase psiCHECK2 construct (see Methods). Next, negative controls for the transfection assays were generated by performing site-directed mutagenesis reactions that resulted in changes of four nucleotides of the respective 3′UTR miRNA-binding sites of *ITGB3* and *CHL1* in the “seed” region (presented in Figure 4A).

*ITGB3* was down-regulated by miR-221/222 in HEK-293T cells 24 h following co-transfection with either miR-221 or miR-222 in combination with the consensus *ITGB3* 3′UTR construct; this was compared to the mutant *ITGB3* 3′UTR construct co-transfection. The Renilla Luciferase-*ITGB3* activity was reduced to 82 and 77%, respectively, of the negative control Renilla Luciferase activity (Figure 4B). Additional time points and human cell lines were tested (see Supplementary Figure S3).

*CHL1* expression regulation by miR-151a-3p was similarly tested in HEK-293T human cell line. *CHL1* expression was directly down-regulated by miR-151a-3p 24 h following co-transfection with miR-151a-3p in combination with the consensus *CHL1* 3′UTR construct and compared with the mutant *CHL1* 3′UTR construct co-transfection. The Renilla Luciferase-*CHL1* activity was reduced to 64% of the negative control Renilla Luciferas activity (Figure 4B). Additional time points and human cell lines were tested (see Supplementary Figure S3).

### miR-151a-3p increases cell sensitivity to paroxetine via down-regulating *CHL1* expression

For determining how *CHL1* regulation by miR-151a-3p affects the cellular response to SSRI drugs, a human LCL (healthy female donor, code #5000, see Methods) exhibiting low sensitivity to growth inhibition by paroxetine was analyzed (Morag et al., 2011; Oved et al., 2012). This particular LCL was chosen for transfection, since it was previously found to express relatively lower levels of miR-151a-3p (Oved et al., 2012), along with higher levels of *CHL1* (Morag et al., 2011), compared with several other LCLs examined from healthy donors.

**Figure 5:**
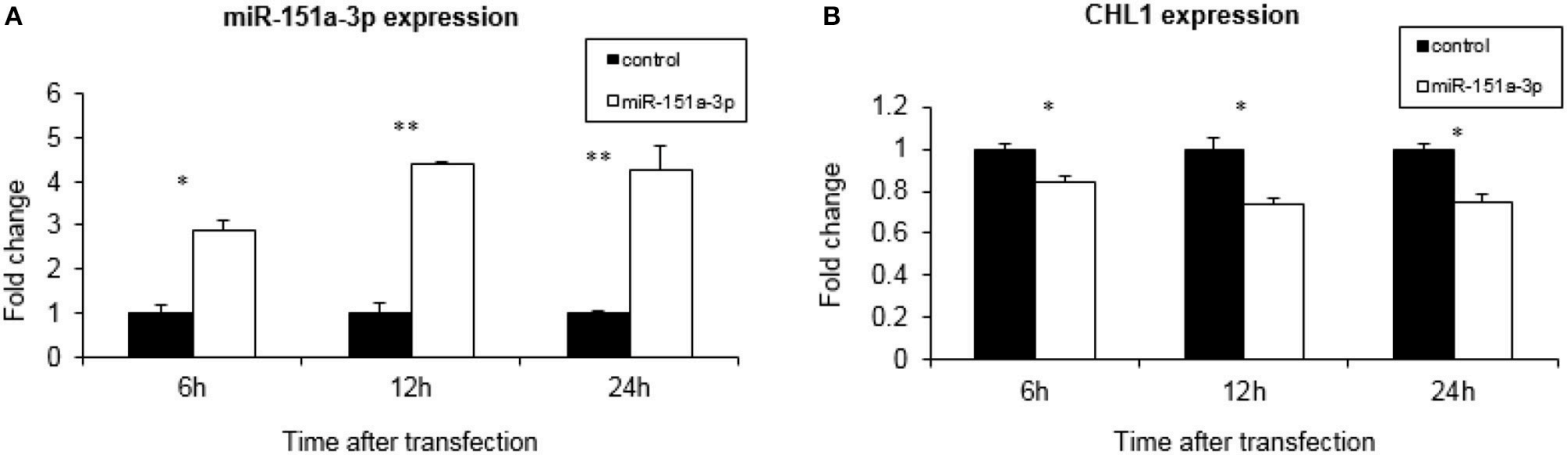
*CHL1* expression affected by miR-151a-3p levels. Real-time PCR analysis of miRNA and gene expression following 6, 12, and 24 h of miR-151a-3p over-expression relative to control plasmid in LCL #5000. The data show the normalized fold-change of **(A)** miR-151a-3p and **(B)** *CHL1*. Values are presented as the mean ± SEM (*n* = 3; ^*^p < 0.05 ^**^p < 0.005).

**Figure 6:**
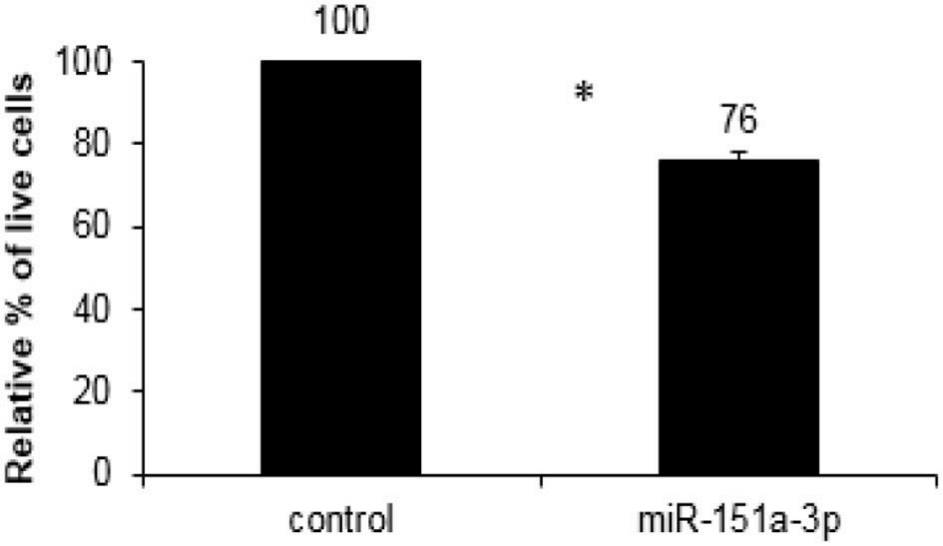
XTT cell viability assay for growth inhibition by paroxetine (10 μM; 24 h exposure) in LCL #5000 following 12 h of over-expression of miR-151a-3p. Values are presented as the mean ± SEM of the relative % of live miR-151a-3p transfected vs. control transfected cells (*n* = 4; ^*^p < 0.05).

We over-expressed miR-151a-3p in this selected LCL and evaluated its expression at different time points following the transfection. miR-151a-3p was significantly elevated by 3–4.5-fold at 6, 12, and 24 h following miR-151a-3p transfection (Figure 5A), along with a corresponding reduction in *CHL1* expression to 74–84% of control transfection values at 6, 12, and 24 h post-transfection (Figure 5B). Moreover, at 12 h following miR-151a-3p transfection, the cell sensitivity to growth inhibition by paroxetine (10 μM) was 24% higher than that of the control transfection (Figure 6).

### Cell adhesion gene expression is increased in serum-free media

**Figure 7:**
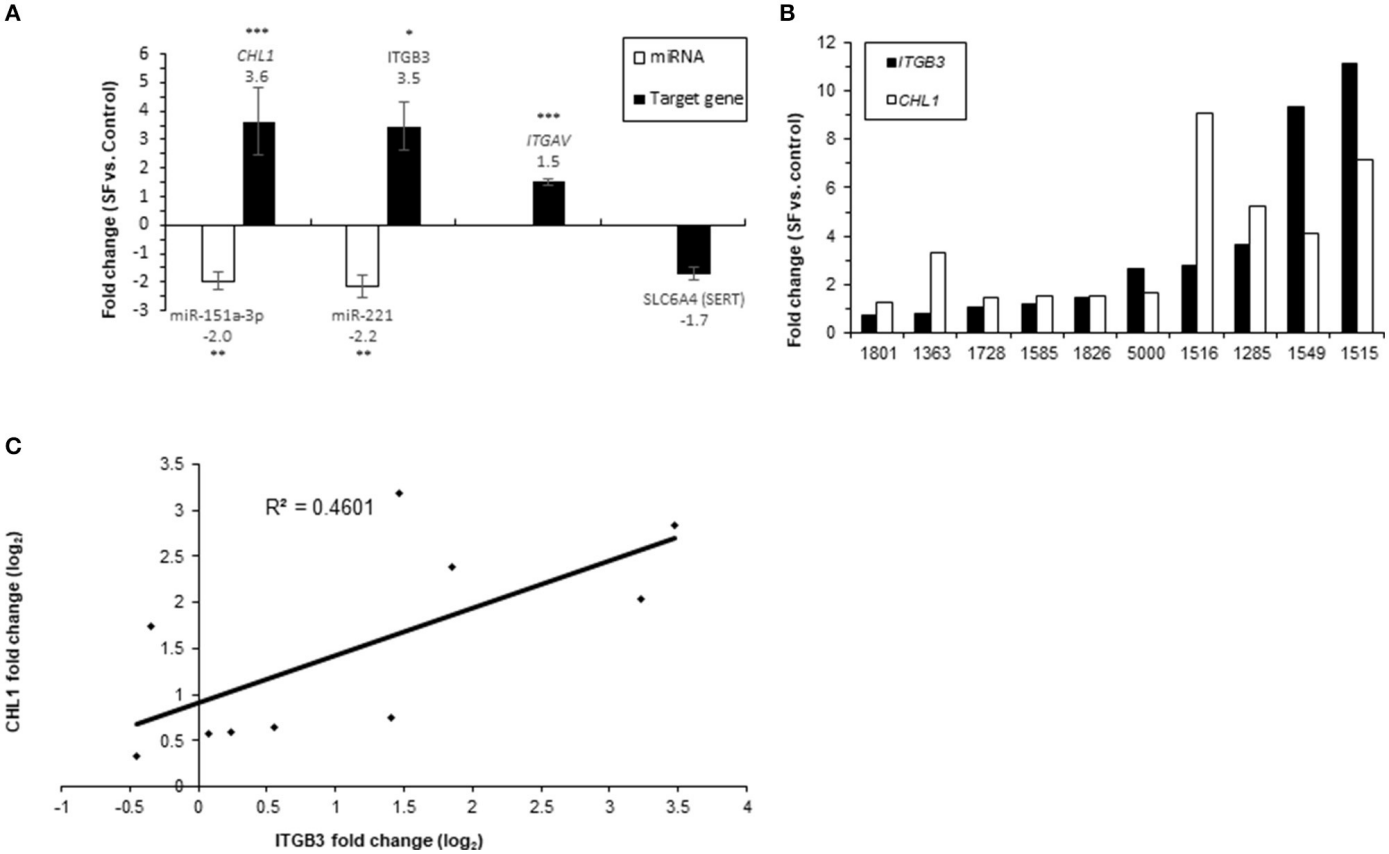
Real-time PCR analysis of miRNAs and gene expression following 21-day growth in SF media vs. serum-supplemented media in 10 LCLs from individual donors. The data show the normalized fold-change of **(A)** miRNAs and their target genes; values are presented as the mean ± SEM (^*^p < 0.05, ^**^p < 0.001, ^***^p < 0.005). **(B)** *ITGB3* and *CHL1* expression for each LCL. **(C)** Correlation plot for CHL1 fold change (log_2_) vs. ITGB3 fold change (log_2_). The numbers on the X axis are LCL donor codes.

The expression levels of SERT (encoded by *SLC6A4*), *ITGAV; ITGB3, CHL1*, and their regulating miRNAs, miR-221 and miR-151a-3p, were compared following 21-day growth of human LCLs from 10 unrelated individuals in serum-free (SF) media supplemented with 4% Biogro-2 (see Methods). LCLs grown in SF media had growth rates and viabilities similar to control cultures grown in serum-supplemented (10% FBS) media, although they divided at a slightly lower rate (not shown). The expression levels of *ITGAV, ITGB3*, and *CHL1* were elevated (average fold-change ± SD of 1.5 ± 0.38, 3.5 ± 2.74, and 3.6 ± 3.71, respectively) following 21-day growth in SF media (Figure 7A). No significant change was observed for *SLC6A4* (SERT) expression levels. Notably, large inter-individual variations between human LCLs from unrelated individuals were observed regarding the extent of up-regulated expression of both *ITGB3* and *CHL1* following 21 days in SF vs. serum-supplemented media, ranging from relatively no change to 11-fold and 9-fold increased expression for *ITGB3* and *CHL1*, respectively (Figure 7B). Moreover, a positive correlation (*R* = 0.68; *P* = 0.031) was observed between the elevated expression levels of these genes following 21 days in SF medium vs. serum-supplemented media (Figure 7C). The respective miRNAs, miR-221, which targets *ITGB3*, and miR-151a-3p, which targets *CHL1*, were down-regulated accordingly following 21 days in SF vs. serum-supplemented media (an average fold change ± SD of −2.2 ± 1.23-fold and −2 ± 1-fold for miR-221 and miR-151a-3p, respectively) (Figure 7A).

## Discussion

Accumulating evidence suggests that the expression of miRNAs is altered during chronic stress, and that dysregulation of miRNAs during such stress may contribute to the etiology and pathophysiology of MDD (Dwivedi, 2011; Mouillet-Richard et al., 2012; Issler et al., 2014; Lopez et al., 2014; Garbett et al., 2015). Moreover, miRNAs may be involved in antidepressant response and may serve as novel antidepressant targets, since different studies have demonstrated altered miRNA expression levels following antidepressant treatment (Bocchio-Chiavetto et al., 2013; O'Connor et al., 2013). Owing to the above considerations and because a single miRNA can potentially regulate scores of mRNAs, miRNAs have the potential to serve as pharmacogenomic biomarkers for SSRI response (Rukov and Shomron, 2011; Labermaier et al., 2013).

Affective disorders, including MDD, which seem to uniquely affect humans (or at least only primates) and are not easily modeled by rodent studies, are related to defects in higher human cognitive processes and to the complexity of the human brain compared with the rodent brain (Geschwind and Rakic, 2013; Czéh et al., 2016). MDD is a complex mood disorder with high comorbidity with anxiety disorders, characterized by psychiatric diagnoses of human behaviors such as sadness, guilt, and feeling of worthlessness, among others. Regulation of gene expression (e.g., by miRNAs) has been suggested to mediate stable adaptations in the brain (Tsankova et al., 2007; Labermaier et al., 2013). Thus, the high level of miRNA conservation among primates observed for our three candidate miRNAs and their respective target gene miRNA-binding sites is intriguing and suggests that an important (and thus evolutionary conserved) role exists for the regulation of *ITGB3* and *CHL1* by miR-221/222 and miR-151a-3p, respectively, in primates (especially in humans) vs. non-primate vertebrates. Moreover, miR-221/222 (to which we will refer together owing to their very close similarity) and miR-151a-3p have a canonical 8-mer site on the 3′UTR region of their targeted genes. Having an 8-mer site was shown to be important for recognition and down-regulation of the target gene (Brennecke et al., 2005; Grimson et al., 2007).

*ITGB3*, coding for the integrin beta-3 subunit, together with integrin alpha-v or with the integrin alpha-IIb, form the vitronectin receptor (αvβ3), and the fibrinogen receptor (αIIbβ3), respectively. Human *ITGB3* has been shown to be regulated by different human miRNAs including miR-98 (Ni et al., 2015), miR-320a (Sun et al., 2015), let-7c (Zhao et al., 2014), and let-7a (Müller and Bosserhoff, 2008). However, in a previous study we did not observe changes in the expression levels of the latter miRNAs following chronic *in vitro* SSRI treatment of human cells (Oved et al., 2013), suggesting that miR-221/222 play a role in the mode of action of SSRI antidepressants.

Using luciferase assays, our present study confirms the direct regulation by miR-221/222 of their potential target-gene, *ITGB3*. In addition, the expression of *ITGB3* was dramatically increased (3.5-fold) following the 21-day growth of human LCLs in SF media, whereas under these conditions, miR-221 was down-regulated in the same cells by 2.2-fold (Figure 7A).

miR-221/222 and their target-gene, *ITGB3*, are expressed in the human brain and are implicated in synaptogenesis/neurogenesis (Cingolani and Goda, 2008; Cingolani et al., 2008; Terasawa et al., 2009; Shao et al., 2010; Hamada et al., 2012; Pozo et al., 2012; Cheng et al., 2014). Using Ilumina sequencing, miR-221 and miR-222 were identified among the top 40 highly expressed miRNAs in post-mortem human brains (Shao et al., 2010); both were induced by nerve growth factor in rat pheochromocytoma PC12 cells (Terasawa et al., 2009; Hamada et al., 2012) and were shown to play a role in neurite guidance (Cheng et al., 2014). Moreover, miR-221 potentiated the formation of neurite networks in these cells (Hamada et al., 2012). ITGB3 is involved in synaptic plasticity in mouse neuronal hippocampal cultures (Cingolani and Goda, 2008) and regulates excitatory synaptic strength (Cingolani et al., 2008) and GluA2 AMPA receptor expression in mouse hippocampi (Pozo et al., 2012).

Importantly, ITGB3 is crucial for maintaining the activity of the serotonin transporter (SERT; encoded in humans by *SLC6A4*), the well-established drug target of SSRI drugs that block serotonin uptake via binding directly to SERT (Sangkuhl et al., 2009). This role of ITGB3 is evident from the diminished SERT activity in platelets (Carneiro et al., 2008), as well as in the midbrain (Whyte et al., 2014) and raphe nuclei synaptosomes (Mazalouskas et al., 2015) of Itgb3-deficient mice, either knockout (*Itgb3*^−/−^) (Carneiro et al., 2008) or heterozygous (*Itgb3*^−/−^) (Whyte et al., 2014; Mazalouskas et al., 2015). Itgb3-knockout mice exhibit altered social and repetitive behavior, e.g., behavior relevant for autism spectrum disorder (Carter et al., 2011). Neuroanatomical assessment of these mice indicated significantly different relative tissue volumes in several brain regions, among them reduced volume of the lateral wings of the dorsal raphe nuclei—a brain region important for the development of the CNS serotonergic system (Ellegood et al., 2012). Moreover, integrins, including ITGB3, interact with CHL1 in the plasma membrane and promote CHL1-induced neuronal migration and neurite outgrowth (Buhusi et al., 2003; Demyanenko et al., 2004; Schlatter et al., 2008; Katic et al., 2014). Considering the above, these findings suggest that ITGB3 plays an important role in correct neuroanatomical development of the CNS, a property it shares with CHL1.

Interestingly, in a pilot clinical study, miR-221 was found to be down-regulated in the plasma of MDD patients treated with the SSRI antidepressant escitalopram (Enatescu et al., 2016). In another study, miR-221 was found to be up-regulated in CSF and serum samples of MDD patients vs. control subjects (Wan et al., 2015). These observations correspond to the decreased expression of miR-221/222 we observed following chronic SSRI treatment in human LCLs (Oved et al., 2013); thus they demonstrate that human LCLs can serve as a legitimate research tool for searching biomarkers for SSRI response in neurons. Whereas in a study that examined blood mononuclear cell proteomes, several members of the integrin signaling pathway, including ITGB3, were found to be differentially expressed between responder and non-responder MDD patients (Martins-de-Souza et al., 2014). In the latter study, ITGB3 expression was found to be increased in responder vs. the non-responder MDD patients.

The identification of *ITGB3* as a potential SSRI response biomarker was further supported by our recent study in which lower *ITGB3* expression levels (~50% vs. healthy controls) were observed in peripheral blood mononuclear cells (PBMCs) obtained from MDD patients (Rzezniczek et al., 2016). The lower *ITGB3* expression levels observed in the PBMCs obtained from the MDD patients is noteworthy and suggests that increased *ITGB3* expression upon chronic SSRI treatment plays a role in the therapeutic action of these drugs in MDD.

Human *CHL1* (close homolog of L1; also known as CALL or L1CAM2) has also been shown to be regulated by additional miRNAs such as miR-10a, miR-590, miR-182, and miR-21 (Long et al., 2012; Chu et al., 2014; Zhu et al., 2014; Li et al., 2016). However, in a previous study we did not observe changes in the expression levels of the latter miRNAs (Oved et al., 2012), suggesting that miR-151a-3p plays a role in the mode of action of SSRI antidepressants.

Using luciferase assays in cells transfected with miR-151a-3p, we confirmed the direct regulation of *CHL1* transcription by this human miRNA, for which the expression levels were observed to be significantly correlated with the *in vitro* paroxetine sensitivity phenotype of LCLs from unrelated healthy individuals (Oved et al., 2012). In addition, the expression of *CHL1* was dramatically increased (3.6-fold) following the 21-day growth of human LCLs in SF media, whereas under these conditions miR-151a-3p, which targets *CHL1*, was down-regulated in the same cells by ~2-fold (Figure 7A). Moreover, following over-expression of miR-151a-3p in human LCLs, we observed a strong reduction of *CHL1* expression (Figure 5B), along with higher *in vitro* sensitivity to paroxetine (Figure 6), as expected from our previous genome-wide transcriptomic study reporting lower *CHL1* expression in human LCLs exhibiting higher *in vitro* paroxetine sensitivity (Morag et al., 2011). Notably, plasma miR-151a-3p was down-regulated in escitalopram-medicated MDD patients (Enatescu et al., 2016).

*CHL1* codes for a cell adhesion protein which plays central roles in neural cell proliferation, migration, differentiation, signal transduction and axon guidance (Maness and Schachner, 2007; Huang et al., 2011). It is implicated in correct brain circuitry (Montag-Sallaz et al., 2002, 2003) and in mental disorders (Frints et al., 2003; Chen et al., 2005). Moreover, *CHL1* expression was downregulated in the hippocampus of mice exposed to early post-natal stress, a known aggravator of mood disorders (Desarnaud et al., 2008). Furthermore, *CHL1/L1* double knockout (*CHL1/L1*^−/−^) mice have misguided neuronal circuitry from the limbic system to the cerebral cortex (Demyanenko et al., 2010, 2011). In addition, *CHL1* was reported to interact with the serotonin 2c receptor and thereby act as a modulator of the serotonergic system (Kleene et al., 2015).

MDD and bipolar disorder are related affective disorders, with BD-II typified mostly by depressive episodes (Baldessarini et al., 2013; Carvalho et al., 2014). Lithium, which is used as a first-line treatment for bipolar disorder, is also employed for augmenting antidepressant therapy in treatment-resistant MDD (Price et al., 1990; Bauer et al., 2003). Notably, lithium was reported to down-regulate miR-221 in the hippocampi of chronically treated rats (Zhou et al., 2009). Moreover, miR-221/222 were down-regulated in the hippocampi of juvenile rats following the induction of lithium-pilocarpine status epilepticus with lithium chloride injections (Ashhab et al., 2013). Taken together, these findings suggest that lithium augmentation of SSRI efficacy in MDD (Price et al., 1990; Bauer et al., 2003) could be related to *ITGB3* up-regulation secondary to miR-221/222 down-regulation.

A recent study by Milanesi et al. reported that the *in vitro* lithium sensitivity of LCLs derived from bipolar disorder patients is affected by IGF-1, an effect that could be observed only in the absence of serum (Milanesi et al., 2015). Notably, IGF-1 was also implicated in MDD (Kopczak et al., 2015). IGF-1 binds directly to integrin αvβ3, and this interaction was found to be essential for IGF-1 signaling through the IGF1R receptor (Saegusa et al., 2009). In addition, IGF-1 assembles the formation of a heterocomplex between IGF1R and the integrin β3 subunit (Tahimic et al., 2016). We therefore compared the transcription of *ITGB3* and *CHL1* in LCLs maintained in SF vs. serum-containing media. In addition, we examined the transcription of *ITGAV*, coding for the integrin alpha-v subunit, which together with integrin beta-3, forms the vitronectin receptor (αvβ3), in SF vs. serum-containing media. The expression levels of *ITGAV, ITGB3*, and *CHL1* were all elevated following 21-day growth of human LCLs in SF media (Figure 7A) and a large inter-individual variation in the extent of up-regulated expression was observed for *ITGB3* and *CHL1* (Figure 7B). A correlation (*R*^2^ = 0.46; *P* = 0.031) was observed between the fold-change of the elevated expression levels of these two genes (Figure 7C). This finding is intriguing as ITGB3 interacts with CHL1 at the cell membrane (Katic et al., 2014), and both *ITGB3* and *CHL1* were implicated in our hypothesized model regarding the mode of action of SSRI drugs (Oved et al., 2013). This model depicts the cell membrane proteins encoded by *CHL1* and *SLC6A4* (coding for the serotonin transporter), competing on a limited cell membrane protein reservoir of integrin beta-3 (encoded by *ITGB3*) (Oved et al., 2013).

Das et al. reported that cultivation of cells in serum-free medium in the presence of fibronectin up-regulates the activity of MMP-2 and MMP-9, two matrix metaloproteinases implicated in cell migration and invasion (Das et al., 2008). Expression of αvβ3 correlates with activation of MT1-MMP and MMP-2 in human melanoma cells (Hofmann et al., 2000). Improved cell adhesion of non-adherent cells in serum-free media has been demonstrated, including for Jurkat lymphoblastoid cells, an immortalized cell line of human T-lymphocytes (Thirumala et al., 2007; Audiffred et al., 2010; Nakayama et al., 2014). These findings correspond to our observations of higher expression levels of the cell adhesion genes *ITGAV, ITGB3*, and *CHL1* in human LCLs maintained in serum-free, compared with serum-supplemented media.

## Study limitations

The present study has several limitations. Although we have previously observed inverse expression patterns between miRNAs and the target gene mRNAs levels in human LCLs and have now experimentally validated miRNA-targets using luciferase reporter assays, one has to keep in mind that our experiments were carried out *in vitro* in human cell lines. We used LCLs from healthy unrelated donors, while the expression of the following miRNAs and target genes may differ in brain tissues of MDD patients. In addition, in order to measure the effect of miR-151a-3p upon SSRI response we used an *in vitro* assay of LCL growth inhibition by the SSRI drug paroxetine as a surrogate for clinical SSRI drug response. Our findings should thus be considered as tentative miRNA/target gene biomarkers for SSRI response until studies with MDD patient blood samples and/or studies with brain tissues of animal MDD models validate our results.

## Conclusions

Taken together, our current observations lend further support for the putative role of miR-151a-3p and miR-221/222 and their (here confirmed) respective target-genes, *CHL1* and *ITGB3*, in SSRI responsiveness, and possibly in the pathology of MDD. We therefore propose that miR-151a-3p and miR-221/222 could tentatively serve as SSRI response biomarkers, as well as potential novel antidepressant therapeutic targets, following additional experimental validation in animal models as well as in clinical trials.

## Author contributions

NS, DG, and KO were responsible for the study design and interpretation of the data. KO, LF, AG, II, and DH were responsible for acquisition of the data.

### Conflict of interest statement

The authors declare that the research was conducted in the absence of any commercial or financial relationships that could be construed as a potential conflict of interest.

## Footnotes

^1^WHO. Depression [Internet]. WHO. [cited 2017 Jul 24]. Available from: http://www.who.int/mediacentre/factsheets/fs369/en/.

^2^miRBase [Internet]. [cited 2017 Jan 19]. Available from: http://www.mirbase.org/

^3^TargetScanHuman 7.1 [Internet]. [cited 2016 Nov 20]. Available from: http://www.targetscan.org/vert_71/

^4^microRNAviewer-all [Internet]. [cited 2017 Jan 19]. Available from: http://people.csail.mit.edu/akiezun/microRNAviewer/index.html.

^5^UCSC Genome Browser Home [Internet]. [cited 2016 Nov 20]. Available from: https://genome-euro.ucsc.edu/index.html.

^6^Amiel. Homepage [Internet]. Natl. Lab. Genet. Isr. Popul. [cited 2017 Jan 2]. Available from: http://yoran.tau.ac.il/nlgip/.
