# Supplementary material for: MicroRNA-Mediated Regulation of ITGB3 and CHL1 Is Implicated in SSRI Action
Source: Front Mol Neurosci. 2017 Nov 2;10:355. doi: 10.3389/fnmol.2017.00355 (PMC5682014; doi:10.3389/fnmol.2017.00355)

**MicroRNA-mediated regulation of *ITGB3* and *CHL1* is implicated in SSRI action**

Keren Oved, et al. Noam Shomron

Supplementary files

**Figure S1.**

Conserved miRs across representative vertebrate species.

Dots represents conserved nucleotides, red square represents miR mature sequence, blue square represents miR seed region.

1. miR-221 (chr. X position 45746157-45746266 +strand)


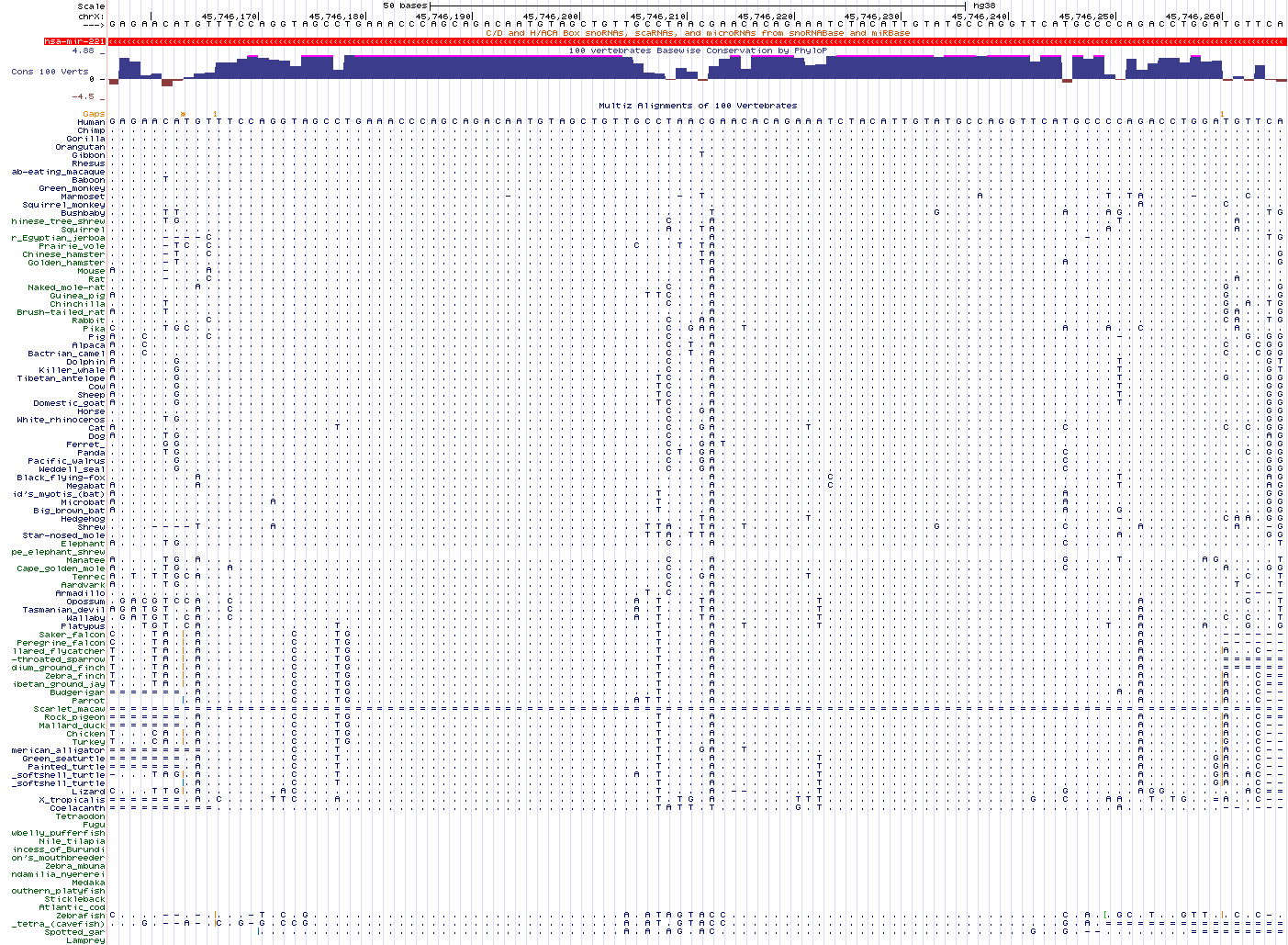


1. miR-222 (chr. X position 45747015-45747124 +strand)
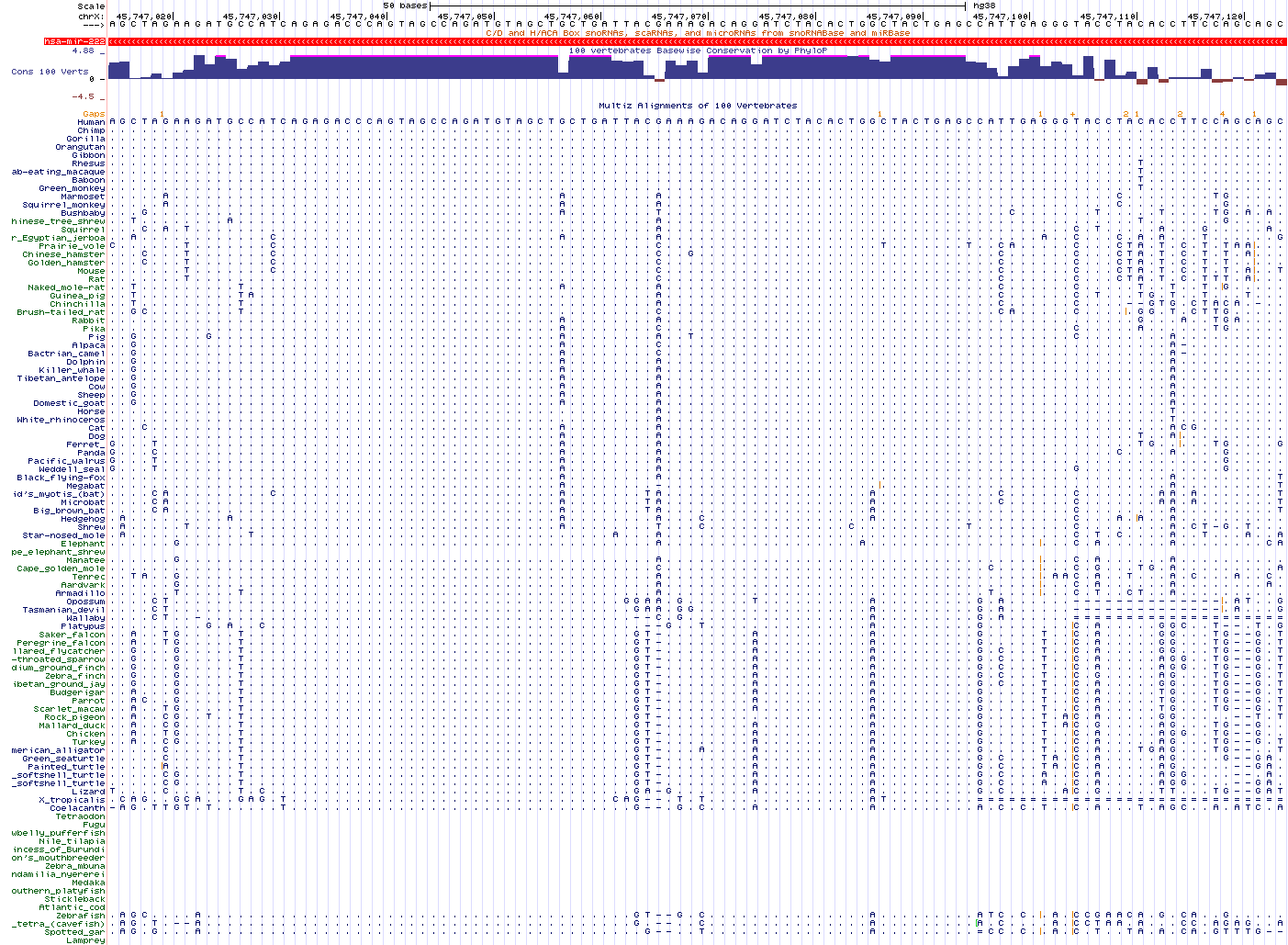

2.
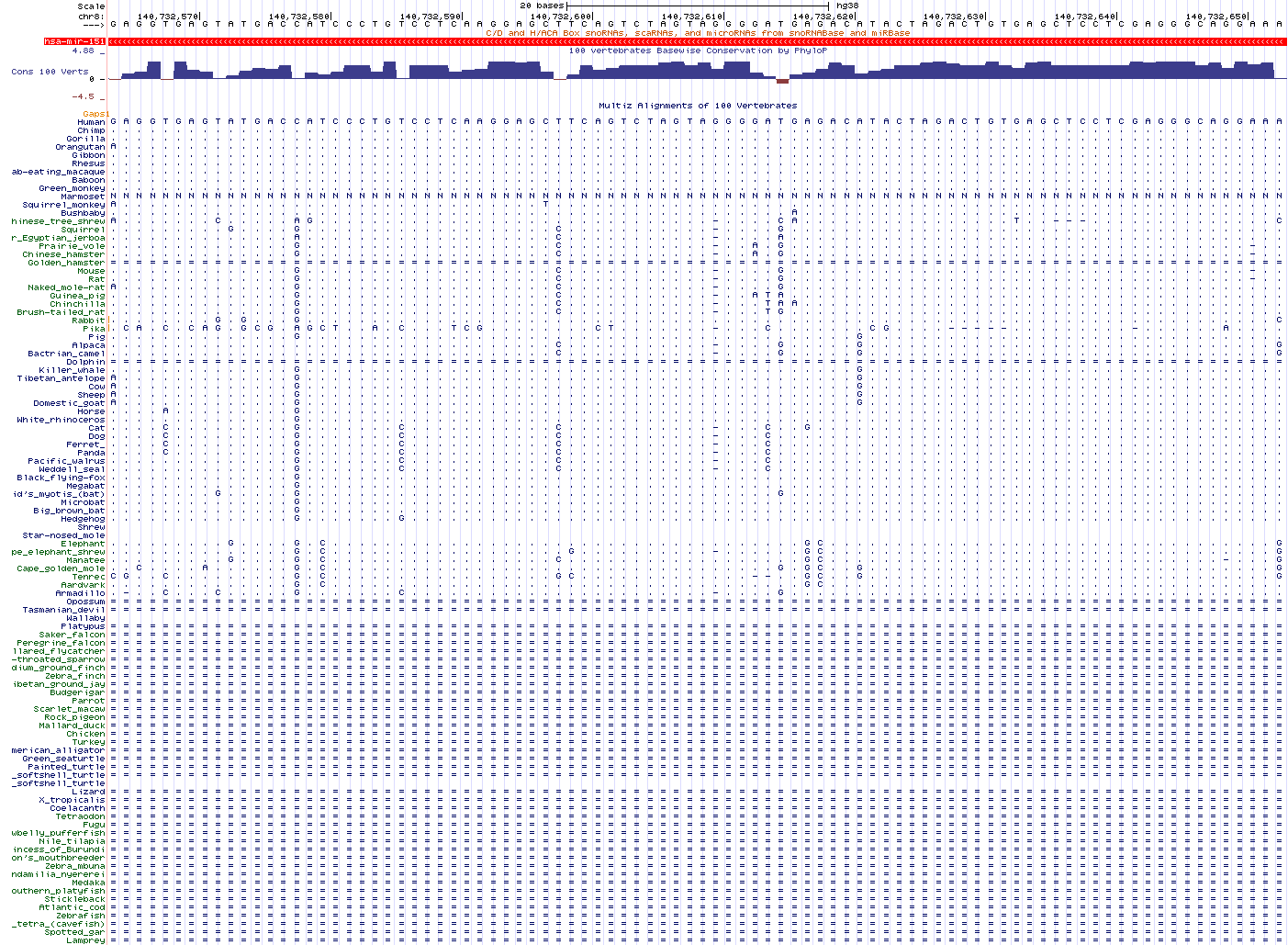
miR-151a-3p (chr. 8 position 140732564-140732653 +strand)

**Figure S2.**

Conserved miRs binding sites across 100 vertebrates.

Dots represents conserved nucleotides, red square represents the seed region + position 8.

1. miR-221/222 target site at *ITGB3* 3’UTR (chr. 17 position 45389536-45389550)


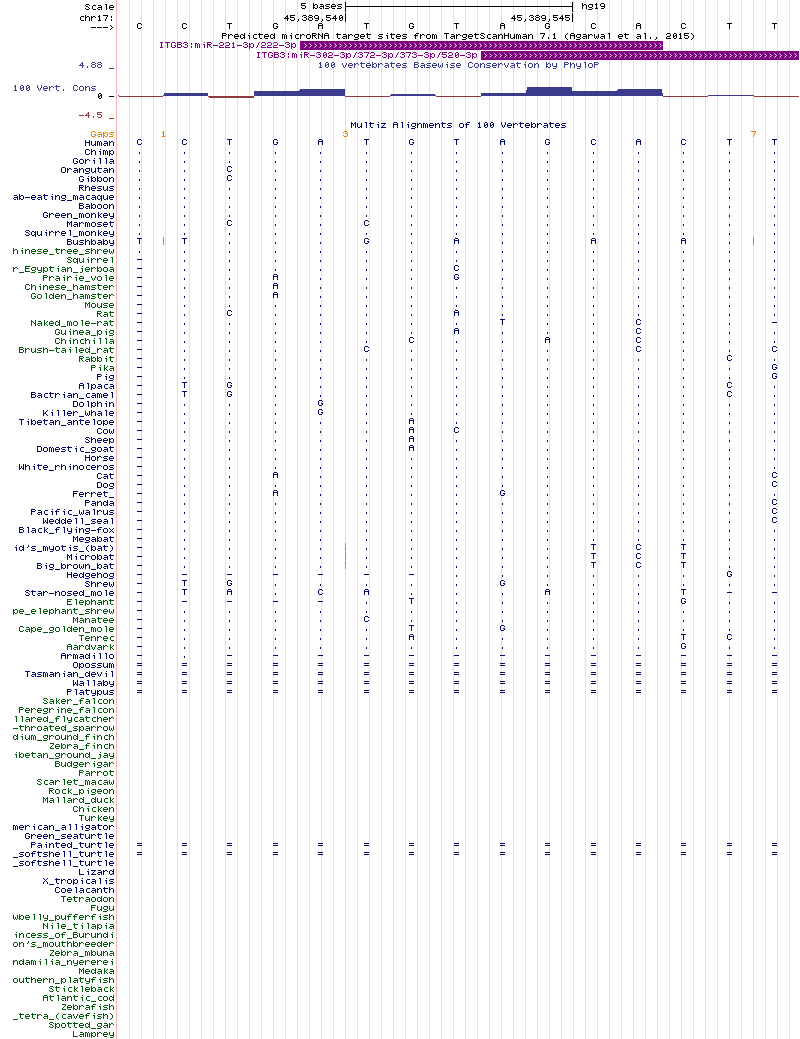


1. miR-151a-3p target site at *CHL1* 3’UTR (chr. 3 position 447894-447908)


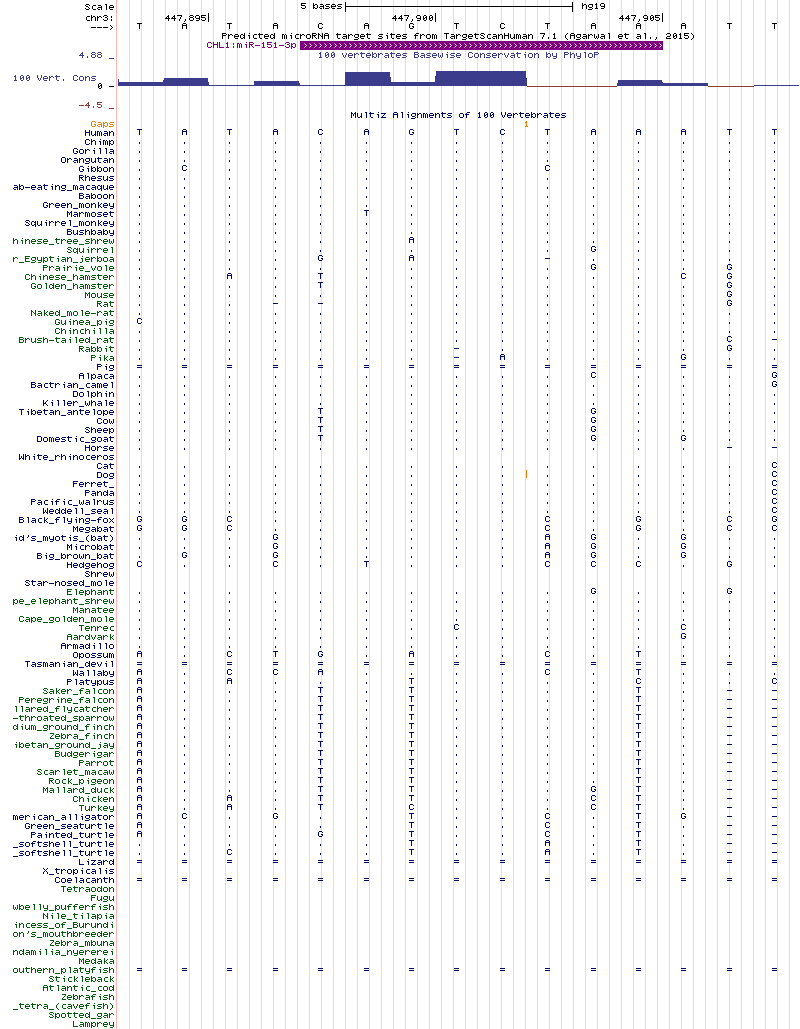


**Figure S3.**

(A) Luciferase activity 24 hours following co-transfection with miR-221, miR-222 or miR-151a-3p combined with either of the *ITGB3* or *CHL1* 3’UTR constructs (WT vs. mutant) using the SH-SY5Y and MCF-7 cell lines. (B) Luciferase activity 48 hours following co-transfection with miR-221, miR-222 or miR-151a-3p combined with either of the *ITGB3* or *CHL1* 3’UTR constructs (WT vs. mutant) using the HEK-293T and MCF-7 cell lines. Values are presented as the % mean ± SEM (n=3 technical replicates; * p<0.05). O.E., Over-Expression.


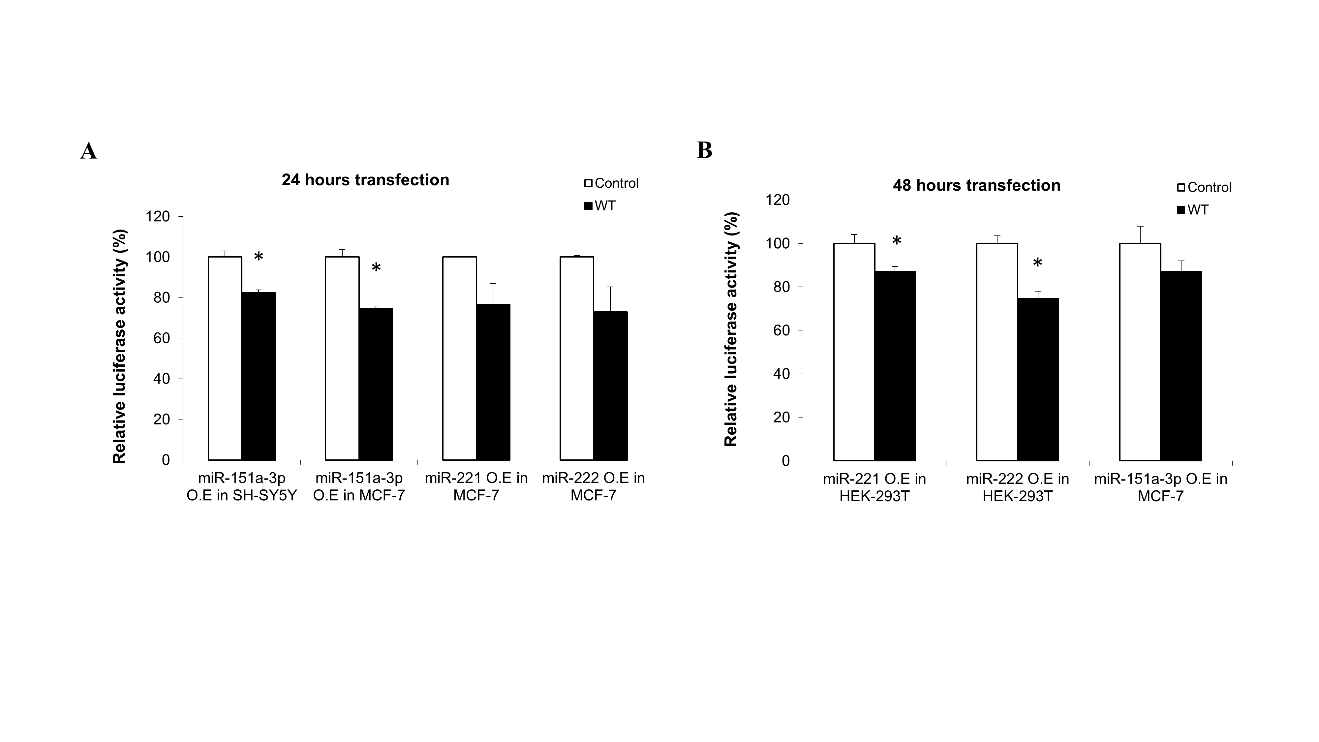

Supplement: Supplementary file 1 [file DataSheet1.DOCX]
